# Polysomnographic Findings in Fragile X Syndrome Children with EEG Abnormalities

**DOI:** 10.1155/2019/5202808

**Published:** 2019-12-03

**Authors:** Marco Carotenuto, Michele Roccella, Francesco Pisani, Sara Matricardi, Alberto Verrotti, Giovanni Farello, Francesca Felicia Operto, Ilaria Bitetti, Francesco Precenzano, Giovanni Messina, Maria Ruberto, Cristiana Ciunfrini, Mariagrazia Riccardi, Eugenio Merolla, Grazia Maria Giovanna Pastorino, Anna Nunzia Polito, Rosa Marotta

**Affiliations:** ^1^Sleep Lab for Developmental Age, Clinic of Child and Adolescent Neuropsychiatry, Department of Mental Health, Physical and Preventive Medicine, University of Campania “Luigi Vanvitelli”, Napoli, Italy; ^2^Department of Psychology, Educational and Science and Human Movement, University of Palermo, Palermo, Italy; ^3^Child Neuropsychiatry Unit, Medicine & Surgery Department, University of Parma, Parma, Italy; ^4^Department of Neuropsychiatry, Children's Hospital “G. Salesi”, Ospedali Riuniti Ancona, Ancona, Italy; ^5^Department of Pediatrics, University of L'Aquila, L'Aquila, Italy; ^6^Pediatric Clinic, Department of Life, Health and Environmental Sciences, University of L'Aquila, L'Aquila, Italy; ^7^Child Neuropsychiatry Unit, Department of Medicine, Surgery and Odontostomatology, University of Salerno, Italy; ^8^Department of Clinical and Experimental Medicine, University of Foggia, Foggia, Italy; ^9^CDR Santa Maria del Pozzo, Somma Vesuviana, Naples, Italy; ^10^Complex Structure of Neuropsychiatry Childhood-Adolescence of Ospedali Riuniti of Foggia, Foggia, Italy; ^11^Department of Medical and Surgical Sciences, University “Magna Graecia”, Catanzaro, Italy

## Abstract

Fragile X syndrome (FXS) is a genetic syndrome with intellectual disability due to the loss of expression of the FMR1 gene located on chromosome X (Xq27.3). This mutation can suppress the fragile X mental retardation protein (FMRP) with an impact on synaptic functioning and neuronal plasticity. Among associated sign and symptoms of this genetic condition, sleep disturbances have been already described, but few polysomnographic reports in pediatric age have been reported. This multicenter case-control study is aimed at assessing the sleep macrostructure and at analyzing the presence of EEG abnormalities in a cohort of FXS children. We enrolled children with FXS and, as controls, children with typical development. All subjects underwent at least 1 overnight polysomnographic recording (PSG). All recorded data obtained from patients and controls were compared. In children with FXS, all PSG-recorded parameters resulted pathological values compared to those obtained from controls, and in FXS children only, we recorded interictal epileptiform discharges (IEDs), as diffuse or focal spikes and sharp waves, usually singles or in brief runs with intermittent or occasional incidence. A possible link between IEDs and alterations in the circadian sleep-wake cycle may suggest a common dysregulation of the balance between inhibitory and excitatory pathways in these patients. The alteration in sleep pattern in children with FXS may negatively impact the neuropsychological and behavioral functioning, adding increasing burn of the disease on the overall management of these patients. In this regard, treating physicians have to early detect sleep disturbances in their patients for tailored management, in order to prevent adjunctive comorbidities.

## 1. Introduction

Fragile X syndrome (FXS) is a genetic syndrome with intellectual disability due to the loss of expression of the FMR1 gene located on chromosome X (Xq27.3) and characterized by an extension of the cytosine-guanine-guanine sequence (CGG) (> 200 copies). This mutation can suppress the fragile X mental retardation protein (FMRP) with an impact on synaptic functioning and neuronal plasticity [1, 2] and direct effect on symptom severity [3–5].

FXS is not limited to the intellectual disability, but organic and behavioral abnormalities are present such as typical dysmorphic facial traits, epilepsy, language disorders, sensory abnormalities with unusual responses to stimuli, motor stereotypes, hyperactivity, attentive problems, executive functioning alteration, coordination skill troubles, and sleep disturbances [6].

On the other hand, among the neurological FXS signs, also EEG abnormalities in the absence of epileptic symptoms seem to be frequent mainly during childhood, consisting of background alterations and interictal epileptiform discharges (IEDs) in different cerebral regions [7–9].

The link between abnormalities in circadian rhythm and IEDs may suggest a common neurochemical alteration in FXS involving the reduction of *γ*-aminobutyric acid (GABA) pathways [10], and enhanced activity of metabotropic glutamate receptors [11], as shown by the data from *Fmr1* knockout (KO) mice [12–14].

Reasonably, these alterations are believed to cause an imbalance leading excitation over inhibition in brain neurophysiology increasing susceptibility to epileptic seizures, IEDs, and sleep dysregulation [15].

Recent studies have shown that FMRP interacts with Kv3.1 mRNA and that dysregulated Kv3.1b translation in a variety of brain regions could be responsible of many symptoms of human FXS. In this sense, Kv3.1b is involved in the auditory brainstem function and in a range of cell types including GABAergic interneurons. Further, the daily fluctuation in Kv3.1b protein levels over the 24 h light/dark cycle seems to be required for circadian neural activity in the pacemaker cells of the suprachiasmatic nucleus [16] and consequently when dysregulated can result in circadian arrhythmicity and probably linked also to mental retardation severity [17].

Consistent with this observation, double knockout mice lacking both *Fmr1* and its homolog *Fxr2* are completely arrhythmic when maintained in normal light/dark cycles [18]. These molecular data highlight that when the fragile X-related proteins are altered or suppressed in their functioning, the circadian rhythm regulation can fail in FXS-affected subjects.

In general, FXS subjects tend to exhibit severe sleep troubles with an estimated prevalence ranging from 31 to 77%, as reported by Kronk et al. [19, 20] with significant sleeping problems, above all difficulty to fall asleep and nighttime awakenings [12, 13]. On the other hand, about 7%-30% of patients present sleep apnea syndrome in comorbidity with FXS [21–25].

About nocturnal overnight polysomnographic studies (PSG), FXS subjects tend to show alteration in REM parameters (i.e., REM reduction and REM latency increase) and reduction in NREM sleep 3 and 4 stages and in total sleep duration [19, 20], associated with an increased motor instability [26] and wake after sleep onset (WASO) increase.

On the other hand, consisting with the sleep regulation pathways, also the sleep microstructure tend to be altered in FXS, with a reduction in low transient EEG oscillations within non-REM sleep [19].

To the best of our knowledge, there are few polysomnographic reports in children and adolescents affected by FXS and with EEG abnormalities. Therefore, the primary endpoint of the present case-control study is to assess the sleep macrostructure, and as secondary endpoint, we evaluate the presence of EEG abnormalities in terms of IEDs in a population of FXS children and adolescents.

## 2. Methods

### 2.1. Study Design

The present research study has been conducted as a multicenter case-control study. Each neuropediatric center recorded at least 1 overnight polysomnographic recordings (PSG), and all recorded data were converted to European Data Format (EDF) in order to permit the PSG scoring independently from the EEG machine acquisition.

### 2.2. Study Population

Twenty-seven children (17 males and 10 females) aged from 6 to 13 years (mean age 9.059 years ± 2.055) with typical FXS genetically confirmed were enrolled in the study and consecutively recruited. EEG abnormalities in terms of IEDs were scored for the nocturnal PSG recordings between May 2015 and September 2018. The PSGs were recorded in the neuropediatric clinical centers.

The control group consists of 27 typically developing children (TDC) (15 males and 12 females, mean age 9.148 ± 2.013), recruited among inpatient subjects (admitted for assessment of recurrent episodes of headache and abdominal pain) resulted negative for neuropsychiatric evaluation and pediatric screening during hospitalization in neuropediatric clinical centers involved in the present study.

Children of both groups are all Caucasians and similar for socioeconomic status. Exclusion criteria were the following for all subjects: genetic intellectual disability syndromes different from FXS (i.e., Down syndrome, Prader-Willi syndrome, and Rett syndrome), autism spectrum disorders (ASD), obesity (*z* − BMI ≥ 95 percentile) and overweight (*z* − BMI ≥ 85 percentile), and epileptic syndromes.

### 2.3. Polysomnographic Sleep Recordings

#### 2.3.1. Sleep Stage Scoring

In the different neuropediatric centers participating to the present study, the PSG montage included a 21-channel digital EEG recording and electrodes are placed according to the international 10-20 system and displayed in a longitudinal bipolar montage; the PSG also included left and right electrooculogram (ROC and LOC), chin electromyogram (EMG), left and right tibialis EMG, electrocardiogram (one derivation), nasal cannula, thorax and abdominal effort, peripheral oxygen saturation, pulse, and position sensors.

Sleep macrostructure, nocturnal respiratory events per hour (apnea/hypopnea index (AHI), oxygen desaturation index (ODI)), and periodic limb movement index (PLMI) were visually scored according to the international standard criteria for pediatric age [27, 28]. An expert scorer (MC) evaluated all the following conventional sleep parameters: time in bed (TIB), sleep period time (SPT), total sleep time (TST), sleep latency (SOL), REM latency (FRL), number of stage shifts/hour (SS/h), number of awakenings/hour (AWN/h), sleep efficiency (SE%), percentage of sleep period time spent in sleep stages 1 (N1%) and 2 (N2%), slow-wave sleep (N3%), REM sleep (REM%), and arousal indexes during the NREM and REM periods. All variables were analyzed by Hypnolab 1.2 sleep software analysis (SWS Soft, Troina, Italy), according to the international criteria for pediatric age.

#### 2.3.2. EEG Abnormalities

An expert pediatric neurologist (AV) evaluated the EEG recordings in order to report a description of the background activity, including details on the posterior dominant rhythm and additional and special features of the background. When detected, IEDs were reported according to their location and distribution, morphology (i.e., spike, sharp, polyspike, and slow-wave), pattern (i.e., single, run, random, rhythmic, and periodic), duration, and incidence (i.e., rare, intermittent, occasional, frequent, and continuous) [29].

### 2.4. Statistical Analysis

Statistical analysis was conducted using STATA/IC version 15. Descriptive statistics were expressed as medians and interquartile ranges (IQR) for continuous variables and as percentages for categorical variables. Comparisons of categorical data were performed with chi-squared test and Fisher's exact test, while continuous data were analyzed with nonparametric Mann–Whitney *U* test. A *p* value ≤ 0.01 was considered statistically significant.

### 2.5. Ethical Approval

Parents of both groups (FXS patients and TDC) gave their written informed consent before the clinical assessment and for the dissemination of results to scientific purposes. The clinical study was conducted according to the principles of the Declaration of Helsinki, and the Departmental Ethics Committee at the Università degli Studi della Campania “Luigi Vanvitelli” approved the study design (Prot. n. 13891 del 9.03.2015; EuDRACT number 2015-001159-66).

## 3. Results

The two groups were matched for age (*p* = 0.873) and sex distribution (*p* = 0.782). The FXS subjects showed significantly lower values compared to TDC for the following PSG parameters: TIB (*p* = 0.0001), SPT (*p* = 0.001), TST (*p* < 0.0001), SE% (*p* = 0.003), and N2% (*p* < 0.0001), while AWN-h (*p* < 0.0001), WASO% (*p* < 0.0001), and REM arousal index (*p* = 0.008) are higher in FXS than in TDC (Table 1).

About respiratory parameters, the AHI (*p* < 0.0001), ODI (*p* < 0.0001), and SpO_2_ desaturation% (*p* < 0.0001) were higher in FXS than in TDC while SpO_2_ nadir % was lower in FXS (*p* < 0.0001) (Table 1). Finally, the PLM analysis showed an index higher in FXS group than in TDC (*p* < 0.0001) (Table 1). No differences were reported between the groups for NREM arousal index, but only for REM arousal index (*p* = 0.001) (Table 1).

In 13 FXS children only, we recorded IEDs, as diffuse (14.814%) and focal spikes and sharp waves (29.629%), usually singles or in brief runs with intermittent or occasional incidence. Different types of IEDs could be recorded also in the same patient.

**Figure 1:**
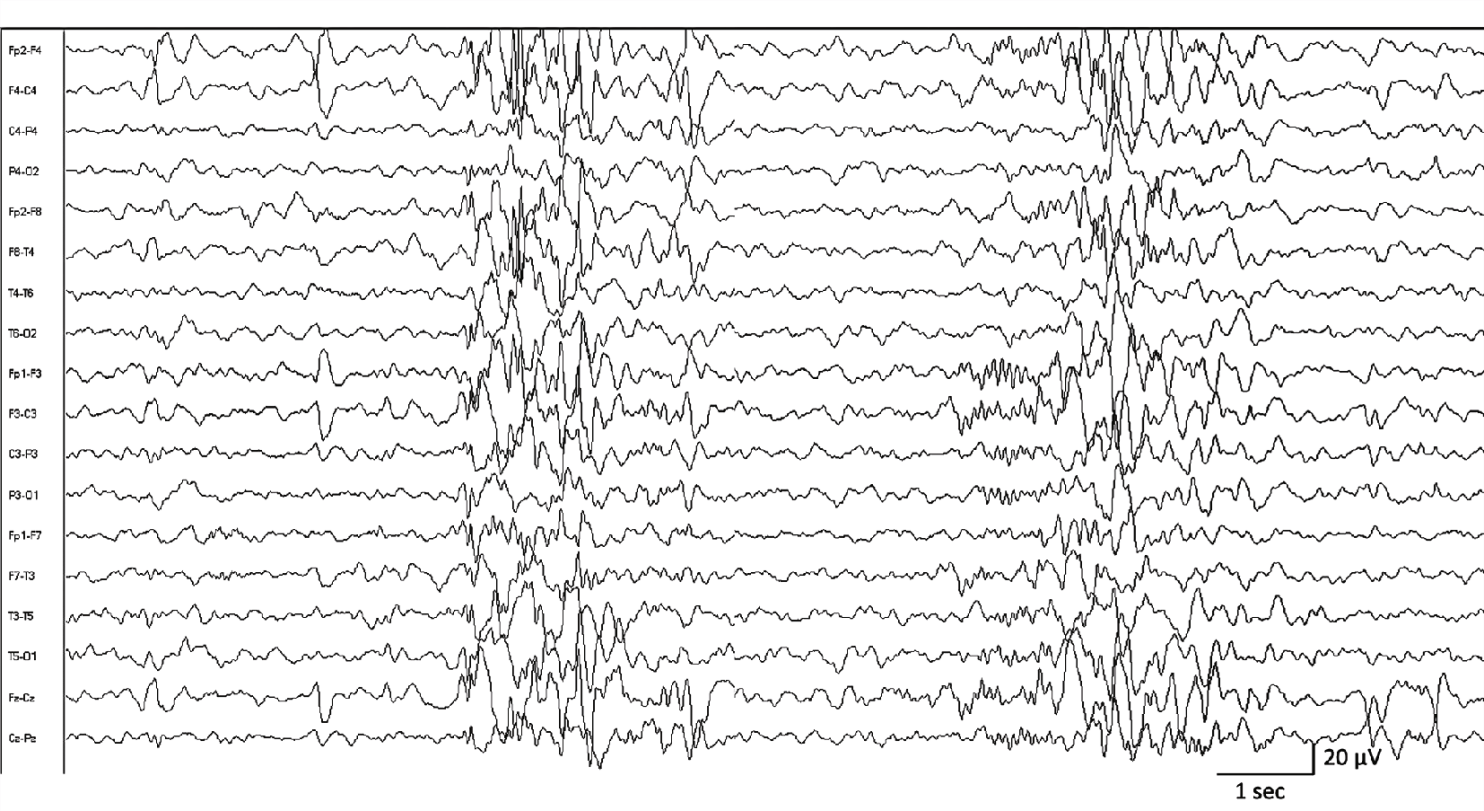
Example of interictal epileptiform discharges (IEDs) during sleep: a 7-year-old boy affected by fragile X syndrome presented a well-organized background activity, and diffuse and irregular runs of interictal epileptiform discharges featuring high amplitude generalized spikes and sharp waves with anterior predominance, sometimes intermingled with the physiological spindles of sleep.

About the FXS subject with IEDs (Figure 1), the two groups with IED and without (no interictal epileptic discharges (NIEDs)) were similar for age (*p* = 0.200), but not for sex because in the IED group, there were 10 males and 3 females while in the NIEDs, female and males were balanced (7 vs. 7). Differences were identified for the PSG findings between the IED and NIED groups in the SPT (*p* = 0.001), TST (*p* = 0.002), and N3% (*p* = 0.001) (Table 2).

## 4. Discussion

The main findings of the present research study can be summarized in the substantial difference in sleep macrostructural parameters between FXS children and healthy subjects. These results may be the putative explanation for the fact that FXS can present alteration in sleep habits [21, 24, 30] impacting cognitive, neuropsychological, behavioral skills [31–34], and parenting quality of life [35].

This syndrome is characterized by a variety of disorders, both physical and behavioral, including sleep disturbances that could be intended as due to alteration in FMR1 gene that alters the sleep-wake cycle [36–38].

However, the pathophysiological mechanisms underlying these alterations are still to be clarified, and it is important to emphasize that such mechanisms do not appear to involve the suprachiasmatic nucleus of the hypothalamus, which is known to play a key role in regulating the circadian rhythm [39, 40].

On the other hand, sleeping and neuronal plasticity are closely linked, also considering that the FMR1 gene plays an important role in the synaptic renormalization of sleep [41]. In 2013, Gonçalves et al. reported that murine models mutated for the FMR1 gene (Fmr1-/- mice) present an abnormality in high synchrony of neocortical network activity, particularly during the first two weeks of life [42]. In this picture, the cortical networks in FXS seem to be hyperexcitable in a brain state-dependent way during a critical period for neuronal plasticity suggesting that these state-dependent network defects may be related to sleep disorders detected in FXS [42]. In addition, KO mice for Fmr1 protein (FMRP) exhibit abnormal circadian behavioral rhythms related to light/dark phases, and the overexpression of FMRP increases PER1- and PER2-mediated BMAL1 (brain muscle aryl hydrocarbon receptor nuclear translocator-like protein 1) and NPAS2 (neuronal Per-Arnt-Sim domain protein 2) transcriptional activity circadian rhythm gene regulators, pinpointing the crucial FMRP role for rhythmic circadian behaviors [18, 43].

Moreover, an association between obstructive sleep apnea syndrome (OSAS) and FXS has been reported. In particular, according to Kronk et al., this association may be reported in about 38-44% of children with FXS [19]. Subjects with FXS exhibit numerous alterations, including facial dysmorphic traits, connective tissue dysplasia, and general muscular hypotonia that are anatomical factors that could increase the risk of OSAS, although the underlying complex mechanisms of this association are not still completely clarified. To explain the higher risk of OSAS also in pediatric subjects with FXS [21], the dysfunction of the autonomic system has been suggested as in other neurodevelopmental disorders [24]. The pathophysiological mechanism underlying this association is still not clear. Studies on animal models have shown that FMR1 gene and the paralog FXR2 are altered in the FXS and play a role in regulating circadian behavior [18, 44–46].

In our sample, FXS children showed pathological values for all nocturnal respiratory parameters (AHI, ODI, SpO2 desaturation%, and SpO2 nadir%) confirming the higher risk for this clinical condition also for OSAS, scarcely reported in current literature. Considering that OSAS may be considered a severe neuroinflammatory condition because of the intermittent nocturnal hypoxia, this condition may also be considered a precipitating factor for FXS neurocognitive symptoms [47–49].

On the other hand, the molecular genetic studies show that expression levels of the circadian gene NR1D2 and CRY2 have reduced in FXS [50]. Few studies exist that specifically address FXS outside of its common comorbid conditions (i.e., ASD) [31], so further research would be needed.

There are few data about sleep problems in people with FXS [38–40]. A study conducted on a sample of 90 affected children, using the questionnaire for sleeping babies (CSHQ) administered to parents, showed that almost half of the children with FXS had clinically significant sleep problems [20]. Another study on a larger sample of 1295 children with FXS showed that in 32% of cases, there was difficulty sleeping, especially difficulty falling asleep and difficulty maintaining sleep [19]. It is also important to consider that FXS can be associated with some comorbidities, which may, in turn, be associated with sleep disorders such as autism [3, 6, 50–52].

The detection of EEG abnormalities, even in the absence of epileptic seizures, has been already described in FXS patients [7–9]. A possible link between these abnormalities and alterations in the circadian rhythm may suggest a common dysregulation of the balance between inhibitory and excitatory pathways in these patients [11–14], leading to increased susceptibility to EEG abnormalities, sleep dysregulation, and epileptic seizures [15]. Moreover, we speculate that the absence of differences in polysomnographic parameters between the group of IED and NIED can be related to the EEG abnormalities; the increase of N3 percentage stage could be due the imbalance in sleep arousal mechanisms related to the disturbances of normal sleep organization, previously reported in FXS [37, 53].

Our study contributes to the currently sparse literature characterizing sleep disturbance in FXS: in particular, it has been previously reported that children suffering from FXS show abnormal sleep patterns [54] and abnormal polysomnographic phenotypes [55] with alteration in sleep microstructure [40]. Moreover, it is important to underline that unlike most of the previous studies, the diagnosis of autism was considered as an exclusion criterion, thus eliminating this possible confounding factor [56, 57]. Considering the strong social and functional impact that sleep disorders can have on children affected by FXS, it is important to evaluate this issue carefully. Further analysis is needed to collect epidemiological data and to understand better the real role of sleep abnormalities as one of the distinctive features of FXS.

## Figures and Tables

**Table 1:** Comparison between fragile X syndrome (FXS) and typical developing children (TDC) for polysomnographic macrostructural parameters. This table shows the differences between fragile X syndrome (FXS) and typical developing children (TDC) for polysomnographic macrostructural parameters: interquartile range (IQR); time in bed (TIB-min); sleep partial time (SPT-min); total sleep time (TST-min); sleep onset latency (SOL-min); first REM latency (FRL-min); stage shifting/h (SS-h); awakenings per hour (AWN-h); sleep efficiency percentage (SE%); wake after sleep onset percentage (WASO%); N1, N2, and N3 stage percentages; arousal indexes during the NREM and REM periods; apnea/hypopnea index (AHI); oxygen desaturation index (ODI); oxygen saturation (SpO_2_); nadir and desaturation percentage levels; and periodic limb movement index (PLMI).

	FXS (*N* = 27)		TDC (*N* = 27)			
	Median	IQR	Median	IQR	*z* value	*p*
TIB-min	497	454-539	584	519-655	-3.797	0.0001^∗^
SPT-min	502	453-522.5	545	485-609.5	-3.157	0.001^∗^
TST-min	409.5	409.5-437	516	462-575	-5.683	<0.0001^∗^
SOL-min	15.2	-27.5-48	19.5	8-37.5	-0.562	0.57
FRL-min	90.5	90.5-90.5	116	81-150	-1.469	0.14
SS-h	7.2	5.3-9.5	8.3	5.3-11.5	-0.839	0.401
AWN-h	7.4	5.6-9.6	1.481	1.5	0.5- 3.2	<0.0001^∗^
SE%	81.261	76.160-90.312	90.1	86.6-94.9	-2.932	0.003^∗^
WASO%	16.505	15.745-17.082	4.7	0.9-7.7	5.701	<0.0001^∗^
N1%	3.027	2.927-3.131	1.4	0.7-4.2	1.549	0.121
N2%	29.760	25.847-31.068	39.5	31.6-44.9	-4.299	<0.0001^∗^
N3%	27.589	26.507-30.130	29.8	24.1-35.9	-0.476	0.634
REM%	23.001	21.078-26.511	21.4	16.2-26.1	1.410	0.15
NREM arousal index	5.644	4.818-6.817	6.645	5.616-7.372	-2.379	0.01^∗^
REM arousal index	9.459	8.858-10.015	8.635	7.824-9.429	2.638	0.008^∗^
AHI	9.889	9.461-10.614	0.448	0.765	0.528-1.069	<0.0001^∗^
ODI	4.139	3.92-4.921	0.469	015-0.863	6.306	<0.0001^∗^
SpO_2_%	93.904	92.156-94.345	98.403	97.889-98.902	-6.306	<0.0001^∗^
SpO_2_ nadir%	89.977	89.148-91.179	96.489	96.282-97.069	-6.306	<0.0001^∗^
SpO_2_ desat%	7.743	7.057-8.468	3.476	2.767-3.862	6.306	<0.0001^∗^
PLMI	6.423	5.256-7.659	2.912	2.393-3.457	6.237	<0.0001^∗^

**Table 2:** Comparison between fragile X syndrome (FXS) with EEG abnormalities (IEDs) and without EEG anomalies (NIEDs) for polysomnographic macrostructural parameters. This table shows the differences between fragile X syndrome (FXS) children with interictal epileptiform discharges (IEDs) and without them (NIEDs) for polysomnographic macrostructural parameters: interquartile range (IQR); time in bed (TIB-min); sleep partial time (SPT-min); total sleep time (TST-min); sleep onset latency (SOL-min); first REM latency (FRL-min); stage shifting/h (SS-h); awakenings per hour (AWN-h); sleep efficiency percentage (SE%); wake after sleep onset percentage (WASO%); N1, N2, and N3 stage percentages; arousal indexes during the NREM and REM periods; apnea/hypopnea index (AHI); oxygen desaturation index (ODI); oxygen saturation (SpO_2_); nadir and desaturation percentage levels; and periodic limb movement index (PLMI).

	FXS IEDs (*N* = 13)		FXS NIEDs (*N* = 14)			
	Median	IQR	Median	IQR	*z* value	*p*
TIB-min	499	459.2-539	493	444.3-535.2	-0.728	0.46
SPT-min	518	505-529	474.75	405-500.5	-3.252	0.001^∗^
TST-min	432.5	419.5-444	411.25	319.5-422	-3.058	0.002^∗^
SOL-min	-1	-59-15.5	35.39	14-69.5	2.135	0.03
FRL-min	90.5	90.5-112	90.5	90.5-114	1.396	0.16
SS-h	6.2	5.3-7.9	9.45	6.2-10.8	1.774	0.07
AWN-h	6.8	5.2-8.3	7.6	5.8-10.5	1.166	0.24
SE%	83.70	80.45-94.36	77.57	71.93-84.15	-1.941	0.05
WASO%	16.50	16.14-16.93	16.52	10.01-17.08	-0.194	0.84
N1%	2.99	2.92-3.06	3.103	2.95-4.625	1.602	0.10
N2%	30.01	29.36-30.79	27.76	23.74-31.06	-0.922	0.35
N3%	26.73	26.15-27.42	29.19	27.67-32.66	3.300	0.001^∗^
REM%	23.74	21.78-25.40	22.29	20.86-27.70	-0.58	0.56
NREM arousal index	5.38	4.79-6.81	5.75	4.83-6.75	0.24	0.80
REM arousal index	9.63	9.01-10.13	9.34	8.38-9.84	-1.165	0.24
AHI	10.23	9.64-10.61	9.76	9.04-10.04	0.922	0.35
ODI	4.32	4.08-4.82	4.05	3.48-4.92	-1.21	0.22
SpO_2_%	94.22	93.53-94.56	92.58	91.87-94.03	-2.23	0.02
SpO_2_ nadir%	90.50	89.65-91.17	89.91	89.13-90.90	-0.776	0.43
SpO_2_ desat%	7.42	6.97-8.46	7.82	7.23-8.24	0.728	0.46
PLMI	5.83	5.06-6.81	6.76	6.01-7.96	1.213	0.22

## Data Availability

The data used to support the findings of this study are available from the first author upon request.
